# Neural Correlates of Feedback Processing in Visuo-Tactile Crossmodal Paired-Associate Learning

**DOI:** 10.3389/fnhum.2018.00266

**Published:** 2018-07-03

**Authors:** Peng Gui, Jun Li, Yixuan Ku, Lei Li, Xiaojin Li, Xianzhen Zhou, Mark Bodner, Fred A. Lenz, Xiao-Wei Dong, Liping Wang, Yong-Di Zhou

**Affiliations:** ^1^Key Laboratory of Brain Functional Genomics (MOE & STCSM), Shanghai Changning-ECNU Mental Health Center, Institute of Cognitive Neuroscience, School of Psychology and Cognitive Science, East China Normal University, Shanghai, China; ^2^NYU-ECNU Institute of Brain and Cognitive Science, NYU Shanghai, Shanghai, China; ^3^Department of Electronic Engineering, East China Normal University, Shanghai, China; ^4^MIND Research Institute, Irvine, CA, United States; ^5^Department of Neurosurgery, School of Medicine, Johns Hopkins University, Baltimore, MD, United States; ^6^Krieger Mind/Brain Institute, Johns Hopkins University, Baltimore, MD, United States

**Keywords:** feedback, ERP, paired-associate learning, visuo-tactile, crossmodal

## Abstract

Previous studies have examined the neural correlates for crossmodal paired-associate (PA) memory and the temporal dynamics of its formation. However, the neural dynamics for feedback processing of crossmodal PA learning remain unclear. To examine this process, we recorded event-related scalp electrical potentials for PA learning of unimodal visual-visual pairs and crossmodal visual-tactile pairs when participants performed unimodal and crossmodal tasks. We examined event-related potentials (ERPs) after the onset of feedback in the tasks for three effects: feedback type (positive feedback vs. negative feedback), learning (as the learning progressed) and the task modality (crossmodal vs. unimodal). The results were as follows: (1) feedback type: the amplitude of P300 decreased with incorrect trials and the P400/N400 complex was only present in incorrect trials; (2) learning: progressive positive voltage shifts in frontal recording sites and negative voltage shifts in central and posterior recording sites were identified as learning proceeded; and (3) task modality: compared with the unimodal PA learning task, positive voltage shifts in frontal sites and negative voltage shifts in posterior sites were found in the crossmodal PA learning task. To sum up, these results shed light on cortical excitability related to feedback processing of crossmodal PA learning.

## Introduction

Establishment of associations between items is of great importance for humans to adapt to dynamically changing environment. For example, getting to know new colleagues relies on associations between their names and appearances, and food selection often relies on the experiential coupling of visual appearances and tastes. These associations may occur within or across sensory modalities, and human beings can quickly acquire such associations either explicitly or implicitly (Miyashita and Hayashi, 2000). A large number of studies in both humans and non-human primates have shown that both “modality specific” sensory areas and association cortices form cortical networks subserving crossmodal associations (Sakai and Miyashita, 1991; Watanabe, 1992; Gibson and Maunsell, 1997; Zhou and Fuster, 1997, 2000; Fuster et al., 2000; Saito et al., 2003; Tanabe and Sadato, 2009; Kassuba et al., 2013; Pillai et al., 2013; Ku et al., 2015; Wang et al., 2015), and with such cortical networks, information about an object can be transferred via cortical associations from one sensory system to another (Calvert, 2001; Fuster, 2001; Bavelier and Neville, 2002).

One of our recent studies (Gui et al., 2017) has demonstrated that middle-stage and late-stage event-related potentials (ERPs; e.g., P400 and a late posterior negative slow wave) during the retention phase of working memory tasks differ between two types of paired-associate (PA) learning (crossmodal vs. unimodal), supporting the notion that the particular neural substrates or neural dynamics are involved in crossmodal working memory and PA learning. In those working memory tasks, participants learned the paired association between stimuli through feedback information in task trials (correct vs. incorrect). Previous studies have revealed that instructive feedback influences performance of PA learning (Jones, 1968; Gagne, 1975). However, it is still unclear how cortical activities (ERPs) related to feedback processing of crossmodal PA learning are modulated during the feedback period of the tasks.

ERP studies exploring the neural dynamics of feedback processing for learning or decision making have generally focused on the utility of the feedback-related negativity (FRN) and the P300. The FRN is commonly computed as the difference in ERP waveform at mid-central recording sites (e.g., the vertex electrode, Cz) between positive feedback and negative feedback, peaking between 200 ms and 300 ms after the feedback onset (Miltner et al., 1997; Nieuwenhuis et al., 2004), which is considered to be driven by prediction error or unexpected feedback (Nieuwenhuis et al., 2004; Bellebaum and Daum, 2008; Pfabigan et al., 2011). Its source is located in brain areas within the salience network (e.g., dorsal ACC, insula) according to observations in an electroencephalogram (EEG)-functional magnetic resonance imaging (fMRI) study (Hauser et al., 2014). The P300, a positive-going ERP deflection roughly peaking around 300 ms after the onset of feedback stimuli (Sutton et al., 1965; Polich, 2007), is related to various task properties such as categorical stimulus probability (Kutas et al., 1977; Johnson and Donchin, 1980), stimulus quality (Smulders et al., 1995), attention level (Polich and Kok, 1995), relevance of task (Squires et al., 1977), complexity of task (Isreal et al., 1980), and the effort required by a task (Brocke et al., 1997). Neural sources of the P300 are less clear, being assumed from a wide range of brain areas, such as the parahippocampal gyrus (Machado et al., 2014), ventral striatum (Pfabigan et al., 2014), areas in the frontal and parietal lobes (He et al., 2001). Although the precise neural origins of P300 and its utility in neuropsychological tests are not clearly known, the P300 is an important signature for cognitive processes, such as attention and working memory (Linden, 2005). Thus, FRN and P300 seem to be related respectively with the salience and early cognitive assessment on feedback stimuli.

Besides the FRN and P300, other ERP components have also been observed from the early stage to the late stage during the feedback period. The P2/N2 complex consists of a frontal positive potential and a posterior N2 potential, which occurs roughly between 180 ms and 300 ms after the onset of stimulus, and this early-stage component has been considered to be related to feature selection and task-relevant stimulus evaluation (Kenemans et al., 1993; Hillyard and Anllo-Vento, 1998; Potts, 2004). The N400, usually elicited by novel or unexpected visual stimuli in linguistic and non-linguistic paradigms, has been thought to reflect a cognitive process on incongruence (Barrett et al., 1988; Koyama et al., 1992; Jemel et al., 1999; Gunter et al., 2000; Finnigan et al., 2002; West and Holcomb, 2002; Ganis and Kutas, 2003; Hagoort, 2003; Olivares et al., 2003), and may be modulated by associative relationships between distinctive stimuli, which are quite independently of semantics (Ortu et al., 2013).

In the cognitive domain, the late potentials (LPs) such as late positive potential (LPP) are thought to reflect sustained attention and cognitive reappraisal on task-relevant stimuli (Schupp et al., 2006). We assumed that the learning process of the crossmodal association was a complex process, which not only required participants to deal with feedback stimuli (positive vs. negative), but also required them to elaborately evaluate the association relationship between the task stimuli (S1 vs. S2) as well. Thus, whether there are ERP components related to feedback of PA learning aside from the FRN and P300, and whether these ERP components differ between crossmodal and unimodal PA learning remain to be elucidated.

**Figure 1:**
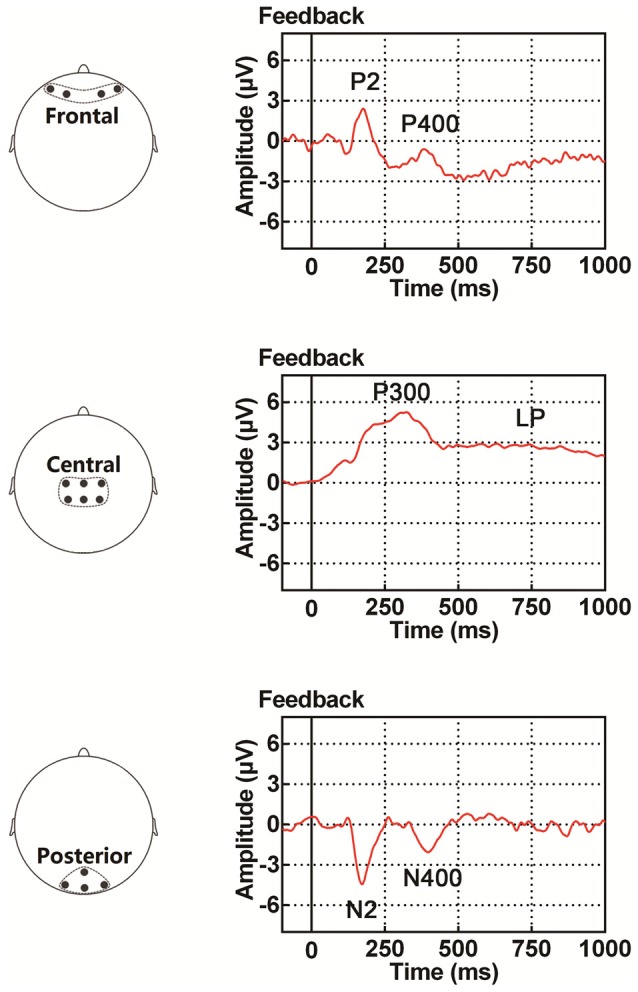
Illustration of event-related potential (ERP) components examined in the present study, including the P2/N2 complex (P2 in the frontal site and N2 in the posterior site), P300, P400/N400 complex (P400 in the frontal site and N400 in the posterior site) and late potential (LP). The time period for the ERP traces is between 100 ms before and 1000 ms after the onset of feedback.

We hypothesized that through examination of the ERP components discussed above, we would likely elucidate the neural substrates of feedback processing in PA learning. Especially, by exploring during the feedback period the difference in scalp voltage between positive feedback (indicating correct responses) and negative feedback (indicating incorrect responses), we might get a better understanding of the effect of feedback type on human brain activity in PA learning. Hence, using scalp EEG recording, the current study aimed at exploring the neural mechanisms underlying feedback processing for PA learning (unimodal and crossmodal) in human participants. EEG data were recorded while participants performed two PA learning tasks, a visuo-tactile (VT) crossmodal task and a visuo-visual (VV) unimodal task. We focused on dynamic changes of four ERP components (P2/N2 complex, P300, P400/N400 complex, LP) during the feedback period of the tasks as illustrated in Figure 1. Specifically, as learning progressed, dynamic ERP changes were examined during the feedback period between PA learning tasks with different modalities.

## Materials and Methods

The protocol of the present study was approved by the Committee on Human Research Protection at East China Normal University (Approval Letter: HR2011/10002). Written informed consent in accordance with the Declaration of Helsinki was given by all participants.

### Participants

Part of the behavioral and EEG data collected from these participants in our lab have been used for another study; Gui et al. (2017).

Twenty-nine right-handed healthy volunteers were recruited in the present study (11 males and 18 females). The average age of the participants was 22.1, with a range from 19 to 26. As measured by the Edinburgh handedness inventory (Oldfield, 1971) and E chart, all participants were with normal or corrected-to-normal visual acuity. None of them had neurological or psychiatric disorders. Nine additional participants were recruited for pre-experimental tests (without EEG recording) to determine whether stimuli and experimental procedures were proper for them to perform PA learning tasks.

### Stimuli

**Figure 2:**
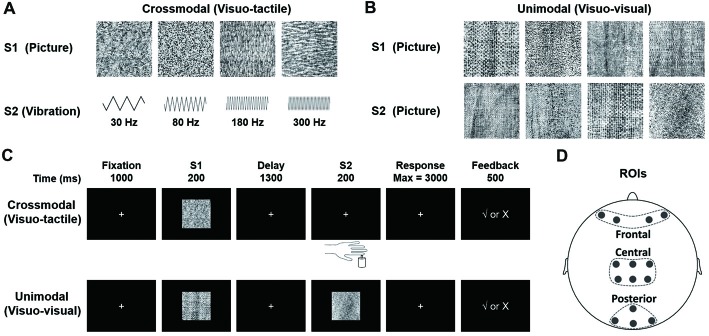
**(A)** Stimuli presented in the visuo-tactile (VT) crossmodal paired-associate (PA) task. Each stimulus (visual or tactile) is presented with a probability of 0.25. In the task, a visual stimulus S1 is followed by a vibrotactile stimulus S2 selected randomly from either a paired one or a non-paired one with a probability of 0.5. **(B)** Stimuli presented in the visuo-visual (VV) unimodal PA task. Each visual stimulus is presented with a probability of 0.25. An S1 is followed by an S2 (either a paired one or a non-paired one with a probability of 0.5). Note that all visual stimuli in this VV task are different from those in the VT task. **(C)** Schematic diagrams showing PA trials. The participant is first presented with one of four visual pictures (S1). After a 1300-ms delay, a second stimulus is presented (S2). S2 is a tactile vibration in the VT task and a visual picture in the VV task. The participant responds with a button press to indicate whether the two stimuli are paired. Visual feedback is presented immediately after the button press to indicate correct/incorrect pairings in learning phases (the first six blocks). **(D)** Electrode placement in frontal, central and posterior areas (Gui et al., 2017).

Experiments were carried out in a quiet room under dim illumination. Visual stimuli used in this study were 12 slides, each of which had a distinctive amorphous texture pattern (Figure 2A upper, 2B), and tactile stimuli were four different frequencies of tactile vibrations (Figure 2A lower). The visual stimuli, obtained from the Internet^1^, were modified to be identical in size (256 × 256 pixels), and to create black-white contrast images. They were presented on a 17-inch CRT monitor (IBM C220P CRT; resolution ratio = 800 × 600 pixels; refresh rate = 60 frames per second) placed 1 m away from participants who seated in a chair facing the screen. The reason for using these pictures as visual stimuli was that they did not have distinguishable features and thus could not be quickly encoded and categorized by the participants. The location of the stimuli was in the center of the screen at participants’ eye level and was within 5° of visual angle. We chose 30, 80, 180 and 300 Hz as frequencies of vibrotactile stimuli, based on equal sensation contours and just noticeable difference for vibrations (Goff, 1967; Pongrac, 2008). The vibration stimuli were delivered to the tip of each participant’s left index finger, which were generated by a permanent magnetic vibrator (LDS V101 vibrator; probe diameter, 6.4 mm) driven by a LDS PA25E Power Amplifier (Brüel and Kjær Sound and Vibration Measurement A/S, Denmark). The amplitude of all vibrations was restricted to the same level (vertical displacement, ±0.049 inches).

Noises accompanying the vibrations were attenuated by using white noise (65 dB SPL) throughout the experiment, which was generated using Adobe Audition 3.0 (Adobe Systems Inc., San Jose, CA, USA) and delivered through two loudspeakers located respectively on each side of the CRT monitor. In addition, the participants wore earplugs to prevent any negative effect caused by white noise. E-Prime 2.0 (Psychology Software Tools, Inc., Sharpsburg, PA, USA) was used for task presentation and behavioral data acquisition.

### PA Learning Tasks

VT and VV PA learning tasks were performed by the participants. Each of the two tasks consisted of nine blocks, and each block consisted of 24 trials. The participants were requested to learn paired associations in the first six task blocks. Intervals of 2 min were set between blocks to reduce participants’ fatigue from task performance. For each task/per participant, 216 trials were collected.

#### Visuo-Tactile (VT) Crossmodal PA Learning Task (Figure 2C, Upper)

At the beginning of each trial, a white fixation cross (2 × 2 cm in size) was presented in the center of the monitor for 1000 ms. It was replaced at the same location by the first stimulus (S1, duration of 200 ms, randomly selected from four pictures with an equal probability of 25%, Figure 2A, upper). The second stimulus (S2), a tactile vibration (randomly selected from four frequencies of 30, 80, 180, or 300 Hz, Figure 2A, lower) with a duration of 200 ms, was presented after a 1300-ms delay. That is, each S1 was followed by a vibrotactile S2 (randomly selected from either a paired one or a non-paired one with a probability of 50%, Figure 2A). The participant was requested to respond to S2 as quickly and accurately as possible by pressing one of the two buttons with their right index finger to point out whether S2 was a paired-stimulus in the trial. Immediately after the choice, a positive (√) or negative (×) feedback sign was displayed. This feedback procedure was only given in the first six blocks, during which the participant was requested to learn paired associations based on feedback information. There was no feedback sign given in the last three blocks. The four pairs of stimuli were randomly defined for each participant before the task began.

#### Unimodal Visuo-Visual (VV) PA Learning Task (Figure 2C, Lower)

The VV task was identical to the VT task except that in this task, instead of a tactile stimulus, S2 was a visual stimulus, a novel one that had never been used for S1 in either the VT task or the VV task (Figure 2B, lower). The order of tasks (VT and VV) and response buttons were arranged in a counterbalanced manner among the participants.

### EEG Recordings and Analyses

All experimental procedures were explained to the participants 1 day before EEG recording. In addition, the participants practiced both unimodal and crossmodal tasks. Each of the tasks contained 24 trials with a pair of stimuli that would not be used in recording experiments carried out on the following day. In the experiment of EEG recording, the participants were instructed to memorize the stimuli only based on visual information but not try to memorize them using auxiliary strategies, such as “naming”, “numbering”, “chunking” or “verbalizing”. For each participant in each task, behavioral and EEG data were both collected from 216 trials (nine blocks). All participants were interviewed about learning strategies they used during the recording experiment right after they completed it.

An EEG recording system (Brain AMP, Brain Products GmbH, Germany) and a 64-channel standard Ag–AgCl electrode cap (64ch-Standard EasyCap placed according to the international 10–20 system, EasyCap GmbH, Germany) were used for data acquisition. EEG activity was online referenced to the electrode at FCz, and offline re-referenced to the common average activity recorded from all EEG electrodes. The impedance of each electrode was kept below 5 kΩ. Electrooculograms (EOG) were recorded from two electrodes to monitor eye blinking and eye movements. The EEG and EOG signals were sampled at 500 Hz.

To more effectively examine feedback-related dynamic changes in both task performance and EEG activity, the first six blocks (out of the total number of nine blocks) were grouped into three sessions for each task (crossmodal and unimodal tasks). They were session “learning I” (block 1 + block 2, crossmodal learning I and unimodal learning I), session “learning II” (block 3 + block 4, crossmodal learning II and unimodal learning II) and session “learning III” (block 5 + block 6, crossmodal learning III and unimodal learning III). An analysis of behavioral and electrophysiological data was conducted over these three sessions. The last two blocks (blocks 8 and 9) were grouped as session “learned” (no feedback in this session). Offline preprocessing of EEG data was implemented in Brain Vision Analyzer 2.0 (Brain Products GmbH, Germany). First, a 0.01–40-Hz band-pass filter was used to reduce the influence of power frequency (50-Hz) and DC drift. Second, an independent component analysis was performed to correct trials with eye blinks and excessive eye movements. Third, trials were rejected if they contained no response to any task event, or contained excessive artifacts in EEG recordings, such as muscle artifacts. Fourth, EEG activity obtained from −100 ms to 1000 ms relative to the onset of feedback in each trial were defined as one epoch. Finally, EEG activity recorded 100 ms preceding the onset of feedback was defined as the baseline, the mean of which was subtracted from the epoch. Topographical maps were drawn over 100 ms intervals for a comparison of scalp voltages under different conditions (feedback type, modality etc.). Fourteen electrodes along the central line were selected and grouped into three distinct regions of interest (ROIs; Figure 2D): frontal site included electrodes of AF3, AF4, AF7 and AF8; central site included electrodes of C1, C2, CP1, CP2, CPz and Cz; and posterior site included electrodes of O1, O2, Oz and POz. The ERP components of interest showed the maximum effect of feedback type, learning and modal at these sites, and EEG signals obtained from the electrodes in each of the ROIs showed highly similar ERP waveforms. The EEG waveforms were arranged in a way that they were: (1) with the same task modality but different feedback types (positive for correct response and negative for incorrect response); (2) with the same task modality but in different learning sessions (learning I, II and III) or (3) in the same learning session but with different modalities (VT and VV). A significant difference in amplitude between two different feedback types was referred to as “feedback type effect”; a significant difference in amplitude among the three learning sessions (learning I, II and III) was referred to as “learning effect”; and a significant difference in amplitude between crossmodal and unimodal tasks was referred to as “modal effect”. Four main ERP components were statistically analyzed. The first component was the P2/N2 complex (peaking around 180 ms after the onset of feedback); the second component was the P300 (peaking around 310 ms after the onset of feedback); the third component was the P400/N400 complex (peaking around 400 ms after the onset of the feedback); the last component was a late slow potential (LP, starting from about 500 ms after the onset of the feedback). Amplitudes of P2/N2, P300 and P400/N400 were respectively measured as an average of 50-ms duration around the peak. The LP, was obtained by averaging EEG activity between 500 ms and 1000 ms after the onset of the feedback.

Depending on their task performance, the participants were categorized into three groups: “Good learner” group (the percentage of correct responses of the last two blocks exceeds 75%), “Poor learner” group (the percentage is lower than 75% in the last two blocks) and “Quick learner” group (the percentage already exceeds 75% in the first two blocks). We set 75% as the cut-off point to classify the participants into different groups following previous studies using learning tasks (DiMattia et al., 1990; Erickson et al., 2005; Gould et al., 2005; McDaniel et al., 2014; Kim et al., 2016). Final results in the current study were only from the “Good learner” group, since sample sizes of the other two groups were not large enough to obtain conclusive results (Supplementary Materials). Two-way repeated measures multivariate analysis of variance (MANOVA) was performed to compare the accuracy as well as reaction time (RT), among learning I, learning II and learning III sessions, and also between the crossmodal and unimodal tasks. Within-participant factors for the analysis were LEARNING (learning I, II and III) and TASK MODALITY (crossmodal and unimodal). Furthermore, three-way repeated measures analyses of variance (ANOVAs) were used to compare amplitudes of ERP components among positions and learning sessions, and between feedback types and tasks. Within-participant factors for these analyses were POSITION (frontal, central and posterior recording sites), FEEDBACK TYPE (positive and negative), LEARNING (learning sessions I, II and III), and TASK MODALITY (crossmodal and unimodal). Two separate three-way ANOVAs instead of one four-way ANOVA were used to examine main effects and interactions of these factors because FEEDBACK TYPE could only be examined during the learning I session but not during the learning II and III sessions due to an insufficient number of incorrect trials collected from those two sessions for meaningful statistical analyses. One of the three-way ANOVAs included the factors of FEEDBACK TYPE, POSITION and TASK MODALITY, and the other included the factors of LEARNING, POSITION and TASK MODALITY. Simple effects analyses were performed if there existed three-way interactions or two-way interactions. Mauchly’s sphericity test was conducted to test the sphericity in repeated measures ANOVA. The Greenhouse–Geisser correction was used if violations to the above statistical assumptions occurred. In the present study, the significance level was set at *p* < 0.05. All statistical analyses were carried out using IBM SPSS 20.0.0 (International Business Machines Corporation., Armonk, NY, USA) or MATLAB 7.11 (The MathWorks, INC., Natick, MA, USA).

## Results

### Task Performance During Unimodal and Crossmodal PA Learning

Nine participants performed pre-experimental tests to examine whether the experimental paradigm would work effectively and whether learning processes were comparable between tasks with different modalities (data not shown). All of them reported that they were able to discriminate one stimulus from another without much difficulty in both crossmodal and unimodal tasks.

Twenty-nine participants performed the VT learning task, and 28 participants performed the VV task (one participant was excluded because of instrument failure). Final results in the current study were only from the “Good learner” group (16 participants who completed the VT task, and 19 participants who completed the VV task). The “Quick learner” group (seven participants for the VT task, and three participants for the VV task) was excluded from subsequent data analyses as the learning effect could not be analyzed due to insufficient learning sessions, and the “Poor learner” group (six participants for the VT task, and six participants for the VV task) was excluded as the sample size was not large enough to ensure an adequate analysis. For the record, the accuracy and RT for each of the nine blocks and for each of the three groups of learners were listed in Supplementary Materials (Supplementary Table S1).

Detailed behavioral results have been shown in our previous study (Gui et al., 2017). Here is a brief outline of those results. The repeated two-way MANOVA analysis showed significant increases in task accuracy (MANOVA, *F*_(1.650,54.439)_ = 230.027, *p* < 0.001, corrected), and significant decreases in RT (MANOVA, *F*_(1.332,43.963)_ = 36.564, *p* < 0.001, corrected) as learning progressed in both crossmodal and unimodal tasks (see Figures 1E–H in our previous article; Gui et al., 2017). The behavioral data from the crossmodal and unimodal tasks were also compared in the repeated two-way MANOVA analysis. The analysis indicated that both tasks were with approximately equal difficulty (MANOVA, accuracy: *F*_(1,33)_ = 3.153, *p* = 0.085; RT: *F*_(1,33)_ = 1.477, *p* = 0.233; Gui et al., 2017). Interviews after the experiment showed that no particular strategy, such as “chunking” or “verbalizing”, was used in either VT or VV task. Participants could also clearly report detailed characteristics of each stimulus and its paired item.

### ERPs Associated With the Effect of Feedback Type

Only ERPs from the first learning session (learning I) were statistically examined for the effect of feedback type, as there were not enough incorrect trials in either the second or the third learning session for a meaningful statistical analysis (Table 1). The average number of correct trials for each individual participant taken for ERP statistical analyses was 29.38 ± 0.90 (mean ± standard error) in the crossmodal task, and 27.16 ± 1.00 in the unimodal task. The average number of incorrect trials for each individual participant taken for ERP statistical analyses was 16.06 ± 0.95 in the crossmodal task, and 19.00 ± 1.08 in the unimodal task. ERP traces from a representative individual participant were also shown in Supplementary Materials (Supplementary Figure S1).

**Table 1:** The average number of trials for each participant in each learning session of the paired-associate (PA) learning task in good learners.

Sessions	Crossmodal task (*n* = 16)		Unimodal task (*n* = 19)	
The total number of trials for each participant in each learning session: 48	The average number of correct trials for each participant	The average number of incorrect trials for each participant	The average number of correct trials for each participant	The average number of incorrect trials for each participant
Learning I	30.87 (64.32%)	17.13 (35.68%)	28.00 (58.33%)	20.00 (41.67%)
Learning II	41.31 (86.07%)	6.69 (13.93%)	37.84 (78.84%)	10.16 (21.16%)
Learning III	43.56 (90.76%)	4.44 (9.24%)	43.47 (90.57%)	4.53 (9.43%)
Learned	44.88 (93.49%)	3.12 (6.51%)	45.21 (94.19%)	2.79 (5.81%)

In parentheses, percentages of trials.

**Figure 3:**
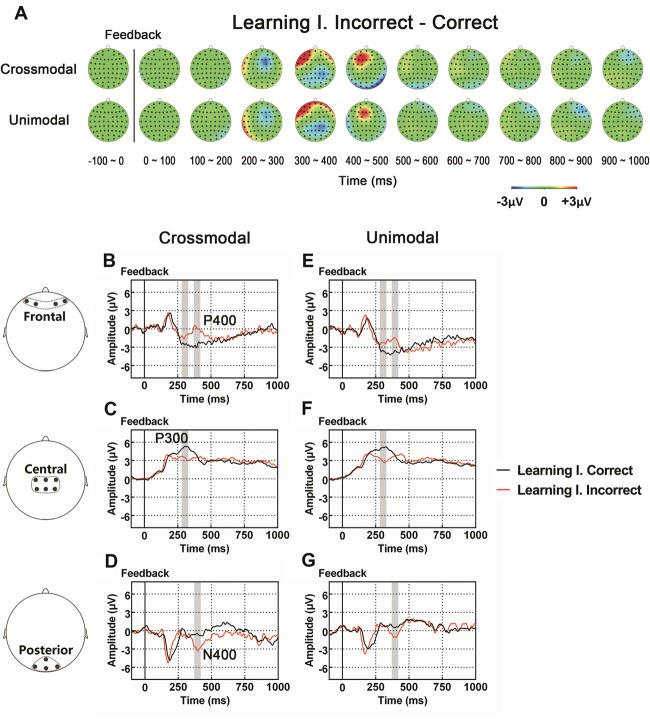
**(A)** Topographic value distribution of whole-brain electrode recordings showing effects of feedback type (correct feedback vs. incorrect feedback) on both crossmodal and unimodal learning tasks for good learners. Values are obtained by calculating voltage differences between incorrect and correct trials during the learning I session, and the time period between 100 ms before and 1000 ms after the onset of feedback is plotted in 100-ms increments. Note that effects of feedback type appear about 400 ms from the onset of feedback as positive voltage shifts around frontal recording electrodes and negative voltage shifts around central and posterior recording electrodes. **(B–G)** Grand average ERPs recorded during the feedback period over frontal **(B,E)**, central **(C,F)** and posterior **(D,G)** recording sites, showing effects of feedback type in both tasks. All ERPs are time-locked to the onset of feedback. Amplitudes of P300 **(C)**, P400 **(B)** and N400 **(D)** significantly differ between trials with different feedback types in both crossmodal and unimodal associative learning conditions. Shaded boxes indicate the time period of the feedback type effects.

As illustrated in whole-brain topographic maps (Figure 3A) and grand average ERP traces (Figures 3B–G), the amplitude of P300 component from central recording electrodes (Figures 3C,F) decreased in incorrect trials. A P400 component from frontal recording electrodes (Figures 3B,E), and an N400 component from posterior recording electrodes (Figures 3D,G) were only observed in incorrect trials. These observations were supported by statistical analyses. Three-way repeated measures ANOVA of the amplitude of P300 (280–330 ms after the onset of feedback) identified significant main effects for POSITION (*F*_(2,66)_ = 48.283, *p* < 0.001) and FEEDBACK TYPE (*F*_(1,33)_ = 10.480, *p* = 0.003). In addition, a two-way interaction between POSITION and FEEDBACK TYPE (*F*_(2,66)_ = 14.090, *p* < 0.001) was observed (Table 2). Simple effects analyses of the P300 were performed at each of the recording sites using two-way repeated measures ANOVA with factors of FEEDBACK TYPE and TASK MODALITY. Results indicated that P300 showed effect of FEEDBACK TYPE in frontal (*F*_(1,33)_ = 16.724, *p* < 0.001) and central (*F*_(1,33)_ = 30.487, *p* < 0.001) recording sites in both crossmodal and unimodal tasks (no significant interaction between FEEDBACK TYPE and TASK MODALITY, *p* > 0.05). Three-way repeated measures ANOVA of the amplitude of P400/N400 complex (370–420 ms after the onset of feedback) identified significant main effect for POSITION (*F*_(2,66)_ = 49.085, *p* < 0.001). In addition, two-way interactions between POSITION and TASK MODALITY (*F*_(2,66)_ = 3.385, *p* = 0.040), and between POSTION and FEEDBACK TYPE (*F*_(1.668,55.045)_ = 19.064, *p* < 0.001, corrected) were observed (Table 2). Simple effects analyses indicated that P400/N400 complex showed effect of FEEDBACK TYPE in frontal (*F*_(1,33)_ = 34.635, *p* < 0.001) and posterior (*F*_(1,33)_ = 15.166, *p* < 0.001) recording sites in both crossmodal and unimodal tasks (no significant interactions between FEEDBACK TYPE and TASK MODALITY, *p* > 0.05). No significant ERP main effect in TASK MODALITY, two-way ERP interaction between FEEDBACK TYPE and TASK MODALITY, or three-way ERP interaction among POSITION, FEEDBACK TYPE and TASK MODALITY was identified by three-way repeated measures ANOVA (Table 2).

**Table 2:** Three-way (TASK MODALITY, POSITION, FEEDBACK TYPE) repeated measures analysis of variance (ANOVA) results for event-related potentials (ERPs) in good learners.

	P2/N2	P300	P400/N400	LP
TASK MODALITY	*F*_(1,33)_ = 1.848 *p* = 0.183	*F*_(1,33)_ = 0.007 *p* = 0.934	*F*_(1,33)_ = 0.145 *p* = 0.706	*F*_(1,33)_ = 0.002 *p* = 0.961
POSITION	***F*_(1.225,40.429)_ = 53.529 *p* < 0.001**	***F*_(2,66)_ = 48.283 *p* < 0.001**	***F*_(2,66)_ = 49.085 *p* < 0.001**	***F*_(2,66)_ = 34.706 *p* < 0.001**
POSITION × TASK MODALITY	*F*_(1.225,40.429)_ = 0.858 *p* = 0.429	*F*_(2,66)_ = 1.930 *p* = 0.153	***F*_(2,66)_ = 3.385 *p* = 0.040**	***F*_(2,66)_ = 3.387 *p* = 0.040**
FEEDBACK TYPE	*F*_(1,33)_ = 0.051 *p* = 0.823	***F*_(1,33)_ = 10.480 *p* = 0.003**	*F*_(1,33)_ = 1.380 *p* = 0.249	*F*_(1,33)_ = 0.461 *p* = 0.502
FEEDBACK TYPE × TASK MODALITY	*F*_(1,33)_ = 0.052 *p* = 0.821	*F*_(1,33)_ = 0.024 *p* = 0.877	*F*_(1,33)_ = 0.083 *p*=0.775	*F*_(1,33)_ < 0.001 *p* = 0.983
POSITION × FEEDBACK TYPE	*F*_(2,66)_ = 0.264 *p* = 0.769	***F*_(2,66)_ = 14.090 *p* < 0.001**	***F*_(1.668,55.045)_ = 19.064 *p* < 0.001**	*F*_(1.671,55.145)_ = 0.889 *p* = 0.400
POSITION × FEEDBACK TYPE × TASK MODALITY	*F*_(2,66)_ = 0.901 *p* = 0.411	*F*_(2,66)_ = 0.108 *p* = 0.898	*F*_(1.668,55.045)_ = 0.548 *p* = 0.550	*F*_(1.671,55.145)_ = 1.077 *p* = 0.338

TASK MODALITY, results for the main effect of task modality in ERPs. POSITION, results for the main effect of position in ERPs. FEEDBACK TYPE, results for the main effect of feedback type in ERPs. POSITION × TASK MODALITY, results for two-way interaction between position and task modality. POSITION × FEEDBACK TYPE, results for two-way interaction between position and feedback type. FEEDBACK TYPE × TASK MODALITY, results for two-way interaction between feedback type and task modality. POSITION × FEEDBACK TYPE × TASK MODALITY, results for three-way interaction among position, feedback type and task modality. Significant statistical results (*p* < 0.05) for the main effect or interaction are in bold.

ERP traces of correct trials and incorrect trials for quick learners (Supplementary Figure S2) and poor learners (Supplementary Figure S3) are shown in Supplementary Materials. Due to an error that occurred during data transfer, EEG data for one poor learner were lost. Thus, only data from five poor learners were included for grand average ERP traces.

### ERPs Associated With the Learning Effect

The learning effect in ERPs was examined among the three learning sessions, as ERPs (P2/N2 complex, P300 and LPs) in those three sessions (the first six blocks) were associated with feedback processing. Only correct trials were used for statistical analyses. In the crossmodal task, the average number of trials for each individual participant in each session was 29.38 ± 0.90 (learning I), 40.00 ± 1.20 (learning II) and 42.31 ± 1.01 (learning III) respectively, and in the unimodal task, the average number of trials in each session was 27.16 ± 1.00 (learning I), 36.79 ± 1.02 (learning II) and 42.47 ± 0.99 (learning III).

**Figure 4:**
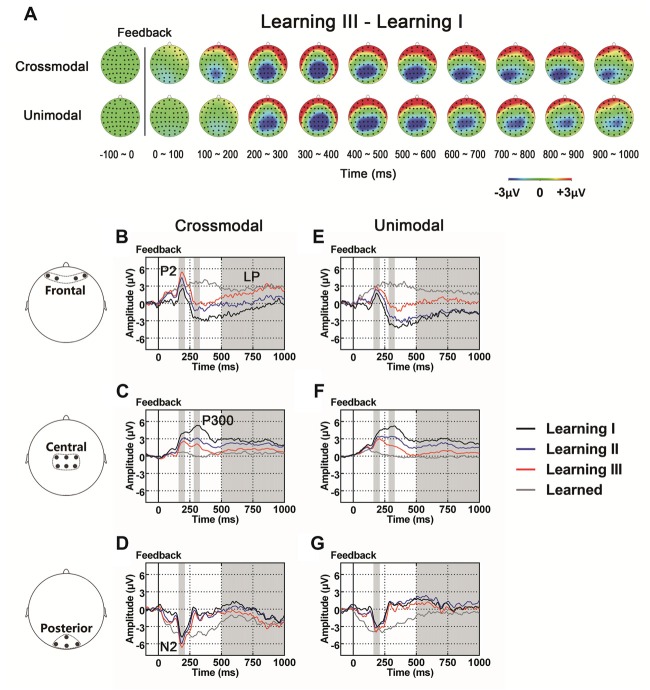
**(A)** Topographic value distribution of whole-brain electrode recordings showing learning effects (learning sessions I, II, III) on both crossmodal and unimodal learning in good learners. Values are obtained by calculating voltage differences between learning I and learning III sessions, and the time period between 100 ms before and 1000 ms after the onset of feedback is plotted in 100-ms increments. Note that learning effects in both crossmodal and unimodal associations appear similar. **(B–G)** Grand average ERPs recorded during the feedback period over frontal **(B,E)**, central **(C,F)** and posterior **(D,G)** recording sites, showing learning effects in both tasks. All ERPs are time-locked to the onset of feedback. Amplitudes of P2 **(B)**, N2 **(D)**, P300 **(C)** and LP **(B)** increase/decrease significantly as both crossmodal and unimodal associative learning processes advance. Shaded boxes indicate the time period of learning effects.

As illustrated in the whole-brain topographic maps (Figure 4A) and grand average ERP traces (Figures 4B–G), positive voltage shifts around frontal recording electrodes and negative voltage shifts around central and posterior recording electrodes were observed in both crossmodal and unimodal associative learning processes. These observations were supported by statistical analyses. Three-way repeated measures ANOVAs revealed significant main effect of POSITION in P2/N2 complex (*F*_(1.324,43.701)_ = 82.656, *p* < 0.001, corrected), P300 (*F*_(2,66)_ = 38.104, *p* < 0.001) and LP (*F*_(2,66)_ = 7.599, *p* = 0.001; Table 3). ANOVA tests also revealed significant main effects of LEARNING in P2/N2 complex (*F*_(2,66)_ = 3.630, *p* = 0.032) and P300 (*F*_(2,66)_ = 11.418, *p* < 0.001; Table 3). In addition, two-way interactions between POSITION and LEARNING were observed in P2/N2 complex (*F*_(2.884,95.187)_ = 10.011, *p* < 0.001, corrected), P300 (*F*_(3.142,103.692)_ = 22.125, *p* < 0.001, corrected) and LP (*F*_(2.464,81.325)_ = 17.233, *p* < 0.001, corrected; Table 3). Simple effects analyses were performed at each of the recording sites using two-way repeated measures ANOVA with factors of LEARNING and TASK MODALITY. Results indicated that P2/N2 ERP complex (160–210 ms after the onset of feedback) and LP (500–1000 ms after the onset of feedback) identified significant effects for LEARNING in frontal (P2/N2: *F*_(2,66)_ = 13.176, *p* < 0.001; LP: *F*_(2,66)_ = 20.100, *p* < 0.001), central (P2/N2: *F*_(1.697,55.992)_ = 12.843, *p* < 0.001, corrected; LP: *F*_(1.498,49.442)_ = 25.374, *p* < 0.001, corrected) and posterior (P2/N2: *F*_(2,66)_ = 5.137, *p* = 0.008; LP: *F*_(2,66)_ = 4.357, *p* = 0.017) recording sites in both crossmodal and unimodal tasks (no significant interactions between LEARNING and TASK MODALITY, *p* > 0.05). Simple effects analyses of P300 identified significant effects for LEARNING in frontal (*F*_(2,66)_ = 17.853, *p* < 0.001) and central (*F*_(1.613,53.240)_ = 55.856, *p* < 0.001, corrected) recording sites in both crossmodal and unimodal tasks (no significant interactions between LEARNING and TASK MODALITY, *p* > 0.05). ERP traces of the learned session (block 8 and 9, the last two blocks of the task) were also plotted in the figure, although they were not taken for statistical comparisons in learning effects, as there was no feedback process in any trial of the session. A comparison was also drawn between the first six blocks and the last three blocks (Supplementary Figure S4).

**Table 3:** Three-way (TASK MODALITY, POSITION, LEARNING) repeated measures ANOVA results for ERPs in good learners.

	P2/N2	P300	LP
TASK MODALITY	*F*_(1,33)_ = 1.441 *p* = 0.238	*F*_(1,33)_ = 1.677 *p* = 0.204	*F*_(1,33)_ = 0.107 *p* = 0.746
POSITION	***F*_(1.324,43.701)_ = 82.656 *p* < 0.001**	***F*_(2,66)_ = 38.104 *p* < 0.001**	***F*_(2,66)_ = 7.599 *p* = 0.001**
POSITION × TASK MODALITY	***F*_(1.324,43.701)_ = 4.547 *p* = 0.029**	*F*_(2,66)_ = 2.480 *p* = 0.092	***F*_(2,66)_ = 3.762 *p* = 0.028**
LEARNING	***F*_(2,66)_ = 3.630 *p* = 0.032**	***F*_(2,66)_ = 11.418 *p* < 0.001**	*F*_(1.622,53.514)_ = 0.154 *p* = 0.813
LEANING × TASK MODALITY	*F*_(2,66)_ = 0.386 *p* = 0.681	*F*_(2,66)_ = 2.372 *p* = 0.101	*F*_(1.622,53.514)_ = 0.295 *p* = 0.700
POSITION × LEARNING	***F*_(2.884,95.187)_ = 10.011 *p* < 0.001**	***F*_(3.142,103.692)_ = 22.125 *p* < 0.001**	***F*_(2.464,81.325)_ = 17.233 *p* < 0.001**
POSITION × LEARNING × TASK MODALITY	*F*_(2.884,95.187)_ = 1.202 *p* = 0.313	*F*_(3.142,103.692)_ = 0.307 *p* = 0.829	*F*_(2.464,81.325)_ = 0.860 *p* = 0.447

TASK MODALITY, results for the main effect of task modality in ERPs. POSITION, results for the main effect of position in ERPs. LEARNING, results for the main effect of learning in ERPs. POSITION × TASK MODALITY, results for two-way interaction between position and task modality. POSITION × LEARNING, results for two-way interaction between position and learning. LEARNING × TASK MODALITY, results for two-way interaction between learning and task modality. POSITION × LEARNING × TASK MODALITY, results for three-way interaction among position, learning and task modality. Significant statistical results (*p* < 0.05) for the main effect or interaction are in bold.

ERP traces of different learning sessions for quick learners (Supplementary Figure S5) and poor learners (Supplementary Figure S6) are shown in Supplementary Materials.

### ERPs Associated With the Effect of Task Modality

**Figure 5:**
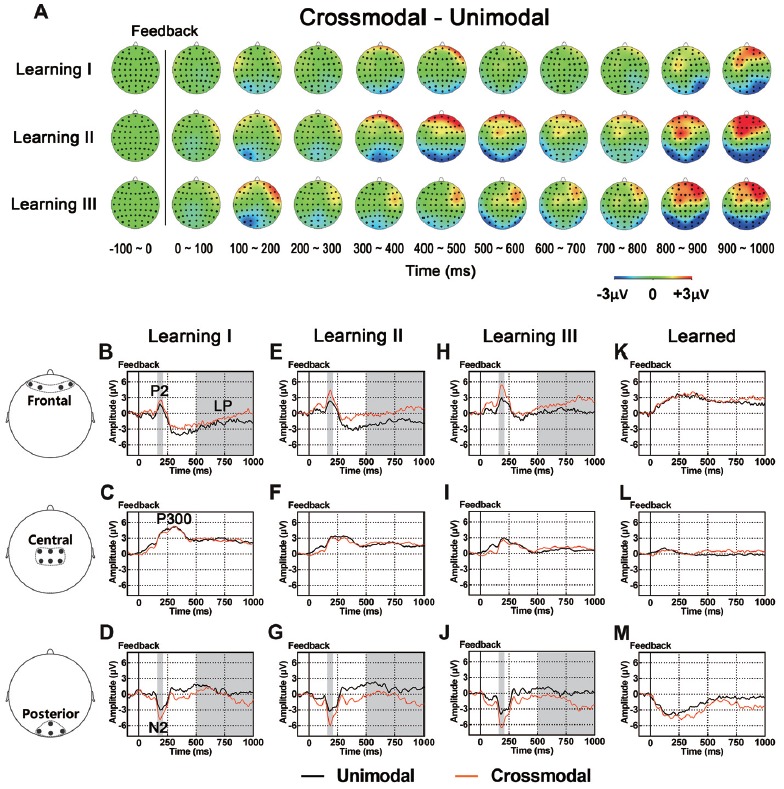
**(A)** Topographic value distribution of whole-brain electrode recordings showing effects of task modality (crossmodal vs. unimodal) in different learning sessions in good learners. Values are obtained by calculating voltage differences between crossmodal and unimodal sessions, and the time period between 100 ms before and 1000 ms after the onset of feedback is plotted in 100-ms increments. Note that modality effects in different learning sessions appear similar: positive shifts in voltage around frontal recording electrodes and negative shifts in voltage around the posterior recording electrodes. **(B–M)** Grand average ERPs recorded during the feedback period over frontal **(B,E,H)**, central **(C,F,I)** and posterior **(D,G,J)** recording sites, showing modality effects in each learning session. All ERPs are time-locked to the onset of feedback. Amplitudes of P2 **(B)** N2 **(D)** and LP **(B)** significantly differ between crossmodal and unimodal associative learning processes. Shaded boxes indicate the time period of task modality effects.

As illustrated in whole-brain topographic maps (Figure 5A) and grand average ERP traces (Figures 5B–M), ERPs around frontal recording electrodes (including P2 and LP) in crossmodal associative learning were more positive than those in unimodal associative learning, and ERPs around posterior recording electrodes (including N2 and LP) in crossmodal associative learning were more negative than those in unimodal associative learning. These observations were supported by statistical analyses. Three-way repeated measures ANOVAs revealed no significant main effect of TASK MODALITY in amplitude of P2/N2 complex, P300 or P400/N400 complex (Table 3). Two-way interactions between POSITION and TASK MODALITY were observed in P2/N2 complex (*F*_(1.324,43.701)_ = 4.547, *p* = 0.029, corrected) and LP (*F*_(2,66)_ = 3.762, *p* = 0.028; Table 3). Simple effects analyses were performed at each of the recording sites using two-way repeated measures ANOVA with factors of LEARNING and TASK MODALITY. Results indicated that P2/N2 ERP complex and LP identified significant effects for TASK MODALITY in frontal (P2/N2: *F*_(1,33)_ = 6.646, *p* = 0.015; LP: *F*_(1,33)_ = 5.172, *p* = 0.03) and posterior (P2/N2: *F*_(1,33)_ = 4.800, *p* = 0.036; LP: *F*_(1,33)_ = 4.466, *p* = 0.042) recording sites (no significant interactions between LEARNING and TASK MODALITY, *p* > 0.05). No significant two-way ERP interaction between LEARNING and TASK MODALITY or three-way ERP interaction among POSITION, LEARNING and TASK MODALITY was identified by three-way repeated measures ANOVA (Table 3). ERP traces of the learned session (block 8 and 9, the last two blocks of the task) were also plotted in the figure, although they were not taken for comparisons here for the same reason mentioned earlier.

ERP traces of different modalities for quick learners (Supplementary Figure S7) and poor learners (Supplementary Figure S8) are shown in Supplementary Materials.

## Discussion

In the present study, we explored the modulation of sequential neural activity associated with feedback processing in crossmodal and unimodal stimulus-stimulus paired-associate learning. Several feedback-evoked ERP components were examined when human participants performed PA learning tasks. Our main findings are: (1) in the feedback period, the P300 decreased with incorrect trials and the P400/N400 complex was only present in incorrect trials; (2) progressive positive voltage shifts were observed in frontal recording sites and negative voltage shifts in central and posterior recording sites as learning proceeded; and (3) positive voltage shifts in frontal sites and negative voltage shifts in posterior sites were observed in the crossmodal PA learning task, compared with the unimodal PA learning task.

Behavioral results showed progressive improvements in task performance (accuracy and RT) in both crossmodal and unimodal tasks during PA learning (for detailed information see our previous study, Gui et al., 2017).

### Components Associated With Feedback Type: the P300 and P400/N400 Complex

Instructive feedback helps learners resolve uncertainty and facilitate learning. Positive feedback informs learners that the current response is correct, and negative feedback informs learners that the current response is incorrect. Neural substrates for positive feedback and negative feedback are different in feedback-based learning (Marco-Pallarés et al., 2007; Bischoff-Grethe et al., 2009; Arbel et al., 2014). Our current data demonstrated that the P300 component in central recording sites and the P400/N400 complex in the frontal and posterior recording sites displayed the effect of feedback type during the feedback processing period in both the crossmodal and unimodal PA learning tasks. These components are likely to represent neural activity associated with the cognitive processing of feedback stimuli.

The P300, an extensively-studied component associated with conscious cognitive processes (Sommer and Matt, 1990; Ridderinkhof et al., 2009), is a positive deflection in voltage to the stimulus with a latency of approximately 300 ms (Sutton et al., 1965; Polich, 2007), which may be generated from a wide range of brain areas (He et al., 2001; Machado et al., 2014). P300 amplitude variation has been indicated to be related to various task manipulations (Kutas et al., 1977; Squires et al., 1977; Isreal et al., 1980; Johnson and Donchin, 1980; Polich and Kok, 1995; Smulders et al., 1995; Brocke et al., 1997). While early studies indicated that the feedback-locked P300 was only sensitive to feedback magnitude but not to feedback type (Yeung and Sanfey, 2004; Sato et al., 2005), recent studies have reported effects of feedback type (San Martín, 2012). Thus, the larger magnitude of P300 for positive feedback observed in our study suggested that positive feedback (a correct response) would raise the level of attention given to active retention of current two stimuli (paired), and the formation of learned associations between the stimuli (Rose et al., 2001; George and Coch, 2011; Steiner et al., 2013; Schomaker et al., 2014; Amin et al., 2015).

The other component observed in the present study was the P400/N400 complex. This complex consisted of a positive deflection in frontal recording sites and a negative deflection in posterior recording sites, peaking near 400 ms after the onset of feedback, and was only observed in trials with negative feedback. To our knowledge, this component has not been reported in previous studies of feedback learning. However, the N400 evoked by visual stimuli has been considered to reflect contextual integration and to be related with semantic processing under incongruent or unexpected conditions (Koyama et al., 1992; Gunter et al., 2000; Finnigan et al., 2002; Hagoort, 2003). Some other studies have shown that there may be a component similar to N400 elicited in non-linguistic paradigms, for instance, when faces or relatively complex pictures are used as a novel or an unexpected stimulus (Barrett et al., 1988; Jemel et al., 1999; West and Holcomb, 2002; Ganis and Kutas, 2003; Olivares et al., 2003). A recent study has suggested that the N400 can be modulated by associative relationships between stimuli, which are most likely not related to semantics (Ortu et al., 2013). In this study, the unrelated target evoked a larger N400 compared with the one evoked by moderate and high association targets. Our observation that P400/N400 complex only appeared in trials with negative feedback, indicated that when negative feedback was presented, this complex likely represented neural activity in brain for updating and memorizing information about the two unpaired stimuli.

### Components Associated With Learning Progress During Feedback: the P2/N2 Complex, P300 and LP

In the present study, a series of ERP components after the onset of feedback showed progressive alterations as learning continued: positive ERP alterations in frontal sites (P2 and LP) and negative alterations in central-to-posterior sites (P300, posterior N2 and LP).

Our findings that the amplitude of P300 and LP in central sites decreased as learning progressed are consistent with those from a previous study (Sailer et al., 2010), in which in contrast to those in control subjects, the feedback P300 and LP were found to decrease in amplitude in participants who had learned the monetary decision-making task. Such results suggested that attentive processing of feedback reduced in the participants. That is, the participants paid less attention on feedback itself once they had acquired the paired association, supporting the view that learning brings about economy of mental effort and more efficient processing.

The posterior N2 evoked by visual stimuli has been considered to be related to feature selection and task-relevant stimulus evaluation (Kenemans et al., 1993; Hillyard and Anllo-Vento, 1998; Potts, 2004), and its amplitude was found to increase with training (Ciesielski and French, 1989; Shen et al., 2006), indicating that, the physiological processes for visual-cognitive tasks become more efficient with practice. The frontal P2 was found to be associated with the attentional process (Carretie et al., 2001; Golob and Holmes, 2011; Buodo et al., 2015). In our current study, the P2/N2 complex is composed of a positive peak in the frontal sites and a negative peak in the posterior sites at around 200 ms after the onset of feedback. Changes in the amplitude of this complex are consistent with those previous studies, suggesting that neural activity represented by this complex is likely involved in early attentional processing of feedback information. A combination of the increase in the amplitude of P2/N2 complex and the decrease in the amplitude of P300 indicates the involvement of those components in the neural dynamics for early attention and other cognitive processes associated with learning during the feedback period.

In the cognitive domain, the LP which begins around 500 ms after the onset of feedback, such as LPP, has been thought to reflect sustained attention and cognitive reappraisal on task-relevant stimuli (Schupp et al., 2006). In the current study, both early-stage (the posterior N2 and frontal P2) and late-stage components (the LPs in frontal and posterior sites) increased in amplitude as learning progressed, indicating that a series of psychological processes occurred in the feedback period, such as the early attentional assessment of feedback stimuli (Hillyard and Anllo-Vento, 1998; Carretie et al., 2001; Potts, 2004; Golob and Holmes, 2011; Buodo et al., 2015), formation of paired associations of two task stimuli (S1 and S2 in crossmodal and unimodal tasks; Kim et al., 2009), the active retention of the stimuli (Schupp et al., 2006).

Previous imaging studies have demonstrated that different brain structures are involved in feedback processing as learning progressed, showing increased activation in the caudate and decreased activation in the left dorsolateral prefrontal cortex (DLPFC; Tricomi and Fiez, 2008). Our observations that progressive temporal changes in scalp voltages occurred as learning proceeded give us a dynamical picture of feedback processing in paired associate learning.

### Components Associated With the Task Modality During Feedback: the P2/N2 Complex and LP

Paired associate memory is related to activity of several brain areas, mainly including the prefrontal cortex, parietal cortex, hippocampus, primary sensory and other association cortices (Mottaghy et al., 1999; Tanabe et al., 2005; Hales et al., 2009; Lee et al., 2016). In human and non-human primate studies, both “modality specific” sensory areas and association cortices have been reported to be involved in crossmodal cortical associations (Sakai and Miyashita, 1991; Watanabe, 1992; Gibson and Maunsell, 1997; Zhou and Fuster, 1997, 2000; Fuster et al., 2000; Saito et al., 2003; Tanabe and Sadato, 2009; Kassuba et al., 2013; Pillai et al., 2013; Zhang et al., 2014; Ku et al., 2015; Wang et al., 2015). With such crossmodal associations, crossmodal associative learning and memory can be achieved as the brain networks supporting the associations are composed of neurons from different cortical areas and therefore information about an object can be transferred crossmodally from one sensory system to another (Calvert, 2001; Fuster, 2001; Bavelier and Neville, 2002). In our previous study (Gui et al., 2017), we found a difference in EEG during the retention period between crossmodal and unimodal working memory tasks, which suggested the involvement of brain structures that processed sensory information in different modalities during that period. In the present study, we explored whether ERPs in the crossmodal task also differed from those in the unimodal task during the feedback period of PA learning. Compared with the unimodal task, a positive alteration in potential in frontal sites (P2 and the LP) and a negative alteration in potential in posterior sites (N2 and the LP) were identified in the crossmodal task across different learning sessions. The difference in ERP between crossmodal and unimodal tasks suggested that the formation of associations between paired stimuli during the feedback period of PA learning was related to sensory modality (modality-specific), and neural networks involved in this formation most likely consisted of different brain regions.

## Conclusion

We examined event-related potentials (ERPs) after the onset of feedback in the tasks for three effects: feedback type (positive feedback vs. negative feedback), learning (as the learning progressed) and the task modality (crossmodal vs. unimodal). ERPs were examined during the feedback period in PA learning tasks, unimodal or crossmodal. We found differences in the amplitude of P300 and P400/N400 complex between positive and negative feedback trials, progressive changes in ERP in frontal, central, and posterior recording sites as learning proceeded, and positive voltage shifts in frontal sites and negative voltage shifts in posterior sites in the crossmodal PA learning task compared with the unimodal PA learning task.

In summary, results in the current study shed light on the temporal dynamics (sequential temporal changes) of neural networks that mediated feedback processing of crossmodal PA learning. Since the precise spatial profile of these hypothesized neural networks could not be directly examined due to the limitation of EEG techniques used in the present study, our future work may use techniques with higher spatial resolution such as electrocorticography (ECoG) and fMRI to investigate how and which particular brain areas get involved in the formation of associations between task stimuli triggered by feedback information.

## Author Contributions

LW, Y-DZ, FL and MB conceived and designed the study. PG and XL developed experimental stimuli. PG and LL collected the data. JL, PG and XZ analyzed the data. JL, PG, Y-DZ, YK, X-WD and LW interpreted the data and wrote the article.

## Conflict of Interest Statement

The authors declare that the research was conducted in the absence of any commercial or financial relationships that could be construed as a potential conflict of interest.

## Footnotes

^1^http://www.zcool.com.cn/gfx/ZMjg3OTI=.html

## Abbreviations

ANOVA: analysis of variance
EEG: electroencephalogram
EOG: electro-oculogram
ERP(s): event-related potential(s)
fMRI: functional magnetic resonance imaging
FRN: feedback-related negativity
LP: late potential
MANOVA: multivariate analysis of variance
PA: paired-associate
ROI(s): region(s) of interest
RT: reaction time
S1: stimulus-1
S2: stimulus-2
VT: visuo-tactile
VV: visuo-visual.
