# Supplementary material for: Neural Correlates of Feedback Processing in Visuo-Tactile Crossmodal Paired-Associate Learning
Source: Front Hum Neurosci. 2018 Jul 3;12:266. doi: 10.3389/fnhum.2018.00266 (PMC6037861; doi:10.3389/fnhum.2018.00266)
Supplement: Supplementary file 1 [file Data_Sheet_1.pdf]

Supplementary Material

Neural correlates of feedback processing in visuo-tactile crossmodal paired-associate learning

Peng Gui*, Jun Li, Yixuan Ku, Lei Li , Xiaojin Li, Xianzhen Zhou, Mark Bodner, Fred A. Lenz, Xiao-Wei Dong, Liping Wang, and Yong-Di Zhou

*** Correspondence:** Corresponding Author: guipeng23@gmail.com or pgui@ion.ac.cn (P. Gui)

1 Supplementary Figures and Tables

1.1 Supplementary Figures

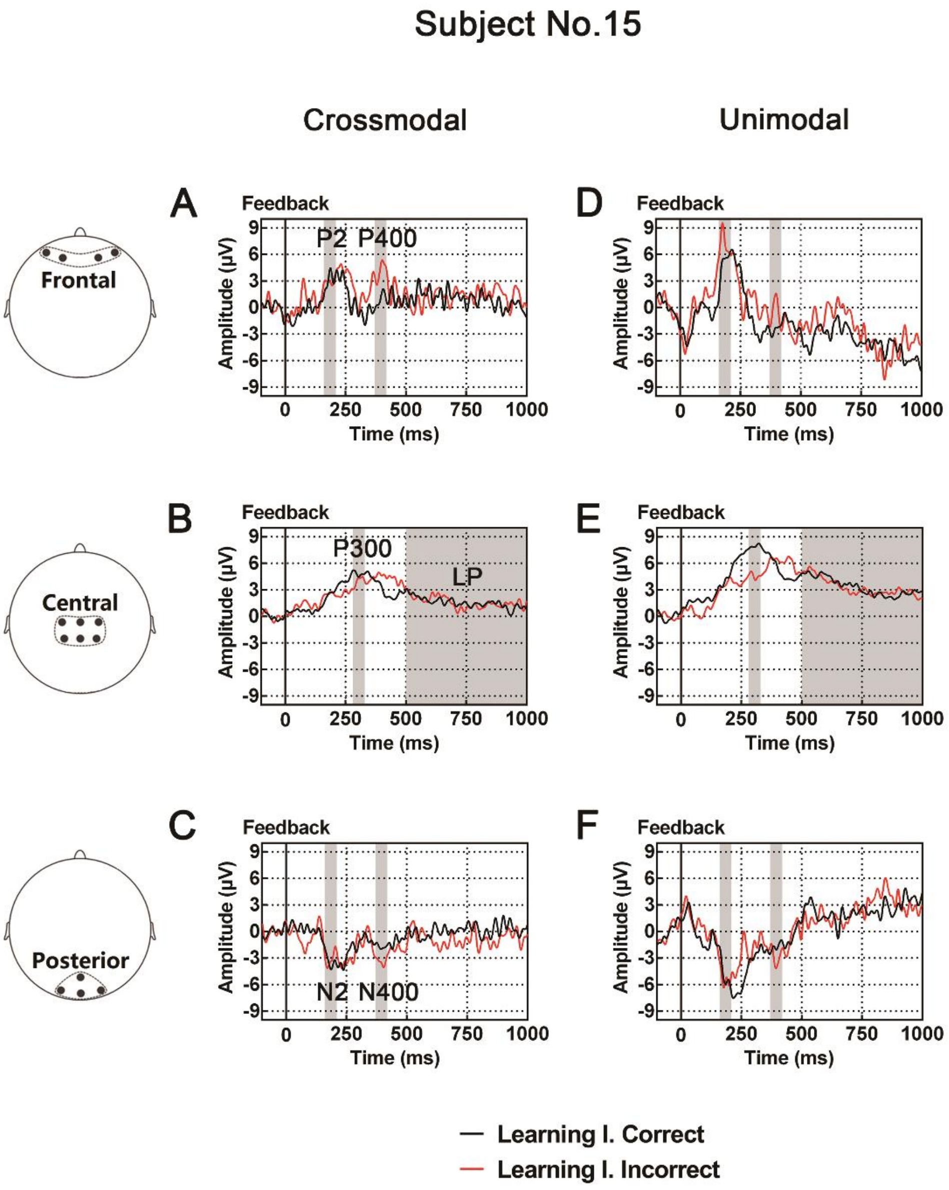

Supplementary Figure 1. Average ERP traces recorded during the feedback period (between 100 ms before and 1000 ms after the onset of feedback) over frontal **(A, D)**, central **(B, E)** and posterior **(C, F)** recording sites for the learning I session of the task in a representative individual participant. ERP components (shaded) for group-level statistical analyses in the present study including P2/N2 complex **(A, C)**, P300 **(B)**, P400/N400 complex **(A, C)** and late potential **(B)** are evident in the participant.

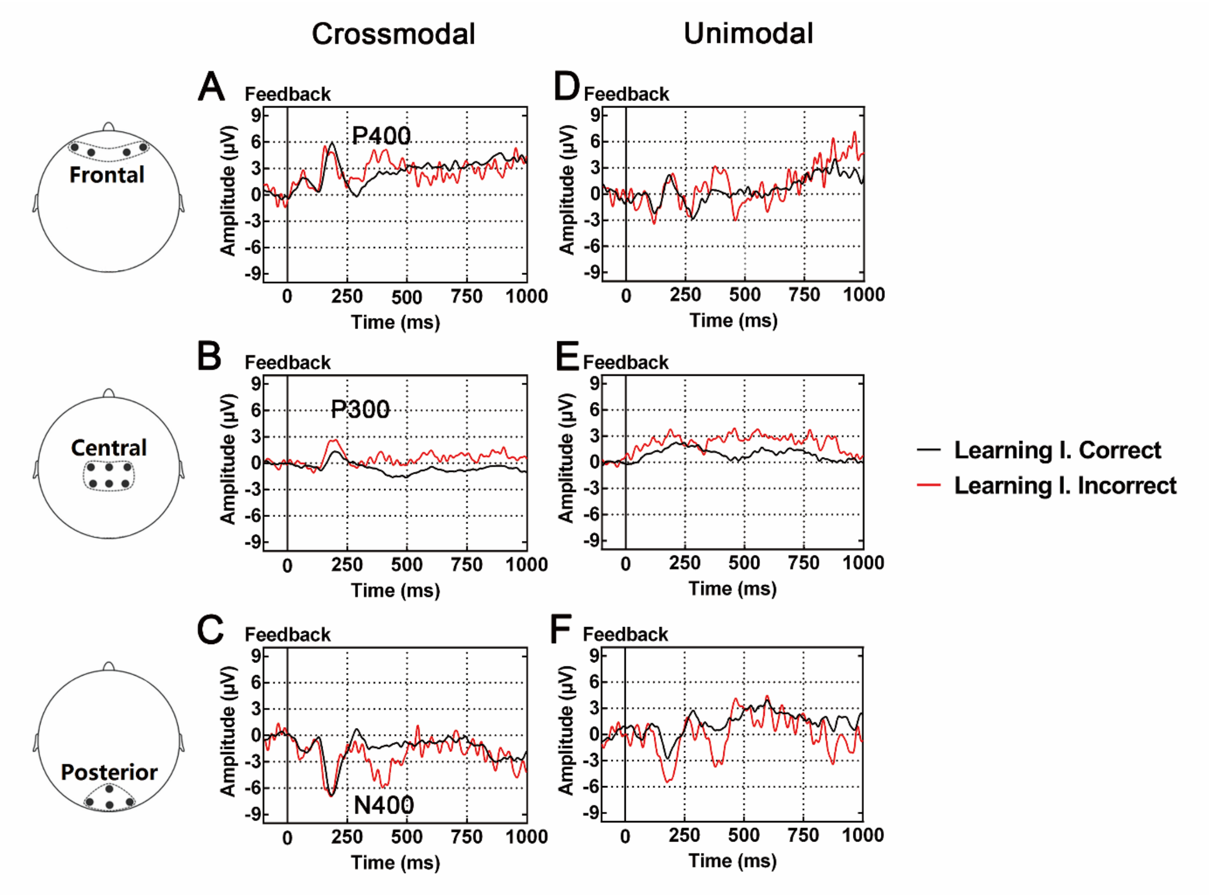

Supplementary Figure 2. Grand average ERPs recorded during the feedback period (between 100 ms before and 1000 ms after the onset of feedback) over frontal **(A, D)**, central **(B, E)** and posterior **(C, F)** recording sites for correct trials and incorrect trials in the quick learners. ERP components including P300 **(B)** and P400/N400 complex **(A, C)** are indicated in the figure.

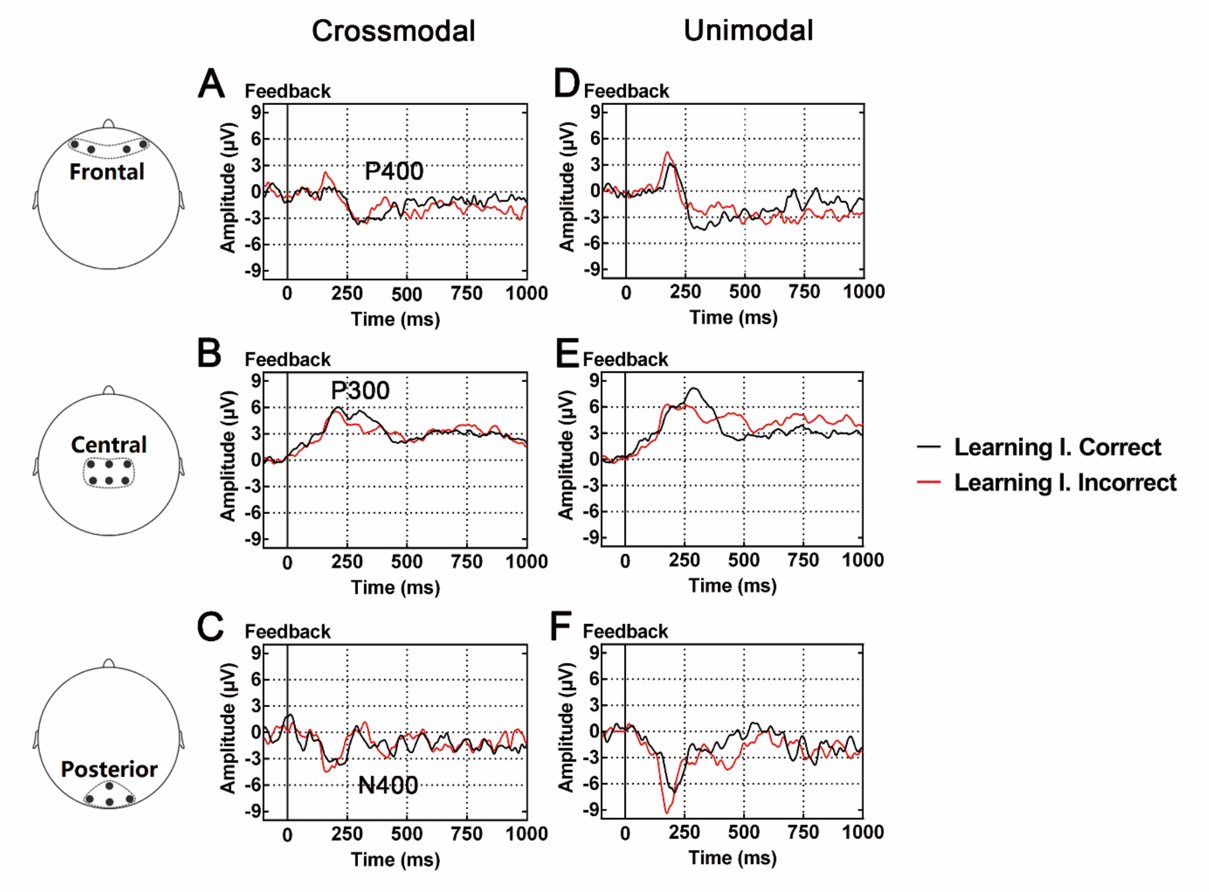

Supplementary Figure 3. Grand average ERPs recorded during the feedback period (between 100 ms before and 1000 ms after the onset of feedback) over frontal **(A, D)**, central **(B, E)** and posterior **(C, F)** recording sites for correct trials and incorrect trials in the poor learners. ERP components including P300 **(B)** and P400/N400 complex **(A, C)** are indicated in the figure.

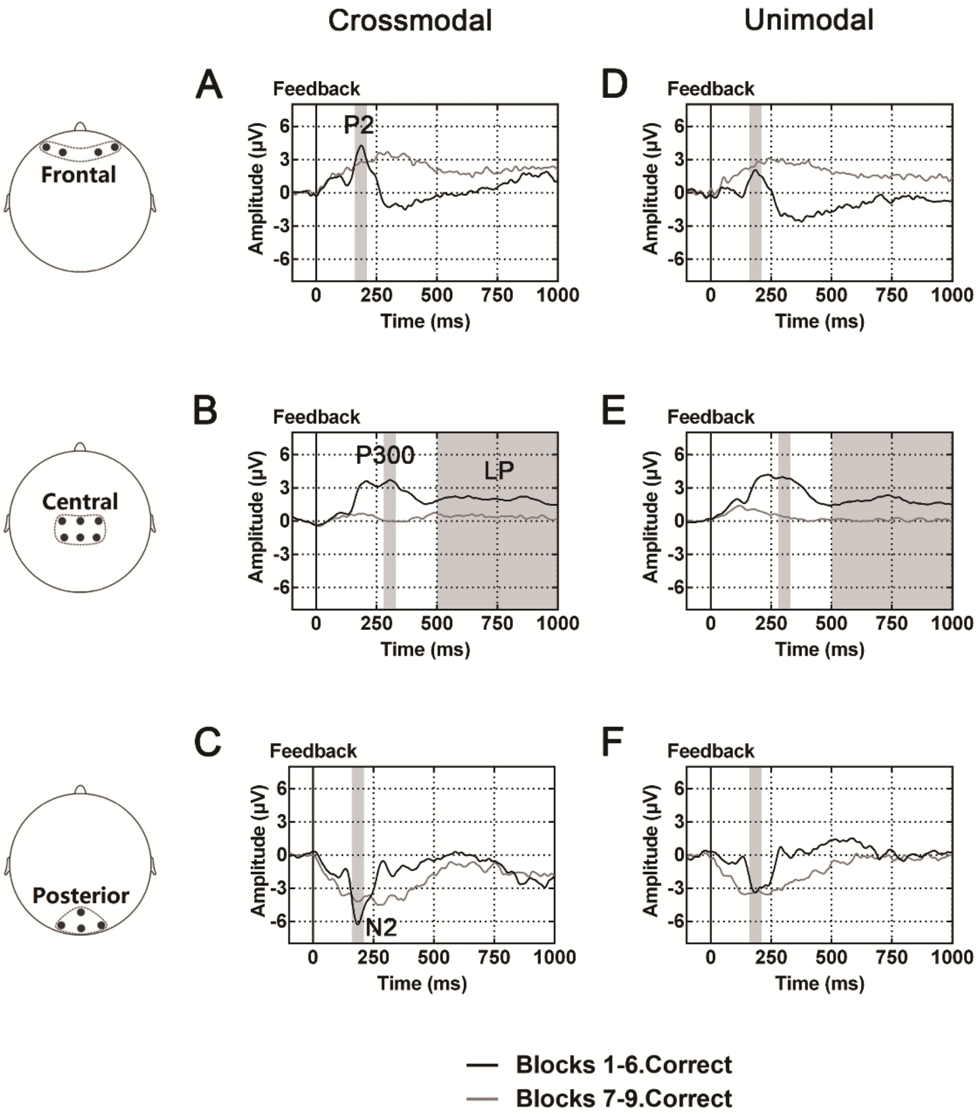

Supplementary Figure 4. ERP traces in the first 6 blocks with feedback and those in the last 3 blocks without feedback in the crossmodal **(A-C)** and unimodal **(D-F)** PA learning tasks. As shown, the ERP components of interest (shaded) including P2/N2 complex **(A, C)**, P300 **(B)** and late potential **(B)** are only evident in the first 6 blocks with feedback.

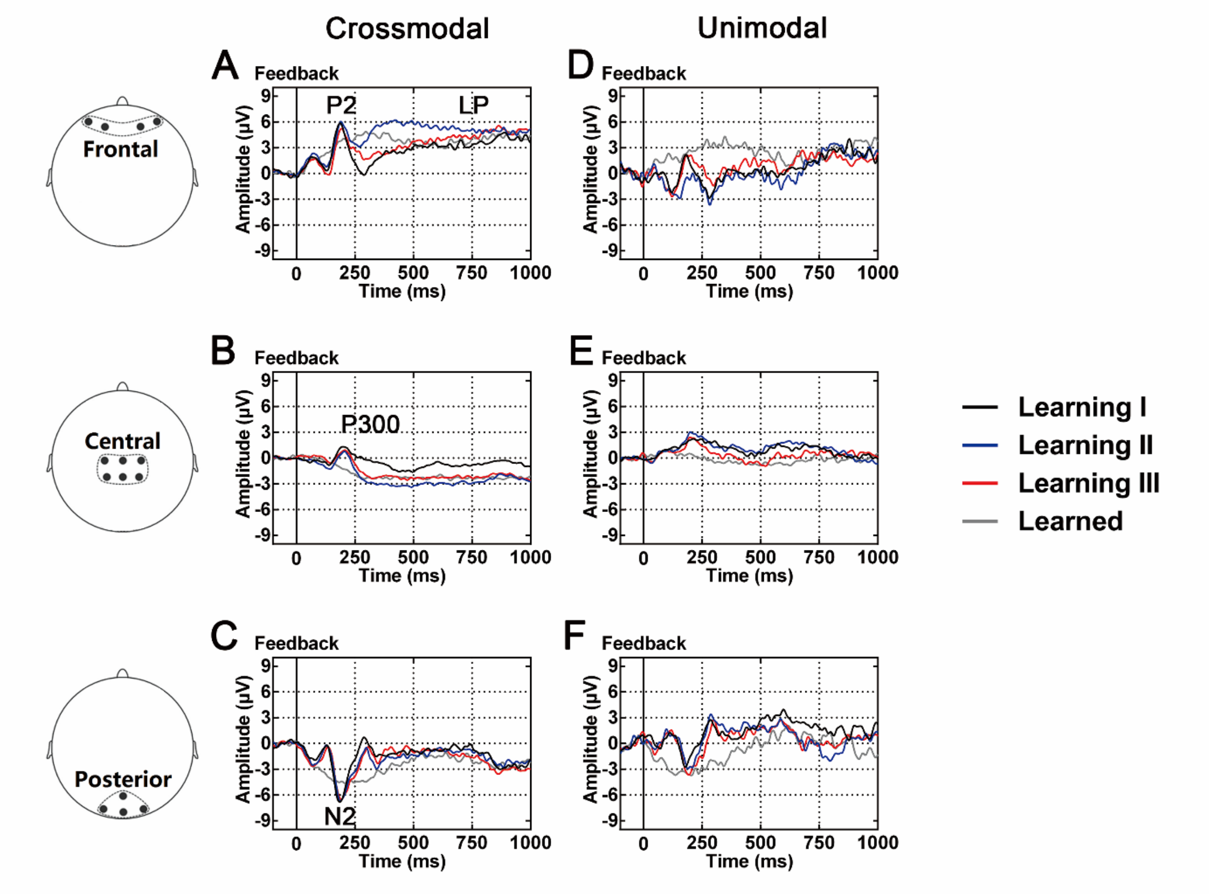

Supplementary Figure 5. Grand average ERPs recorded during the feedback period (between 100 ms before and 1000 ms after the onset of feedback) over frontal **(A, D)**, central **(B, E)** and posterior **(C, F)** recording sites for different learning sessions in the quick learners. ERP components including P2/N2 complex **(A, C)**, P300 **(B)** and late potential **(A)** are indicated in the figure.

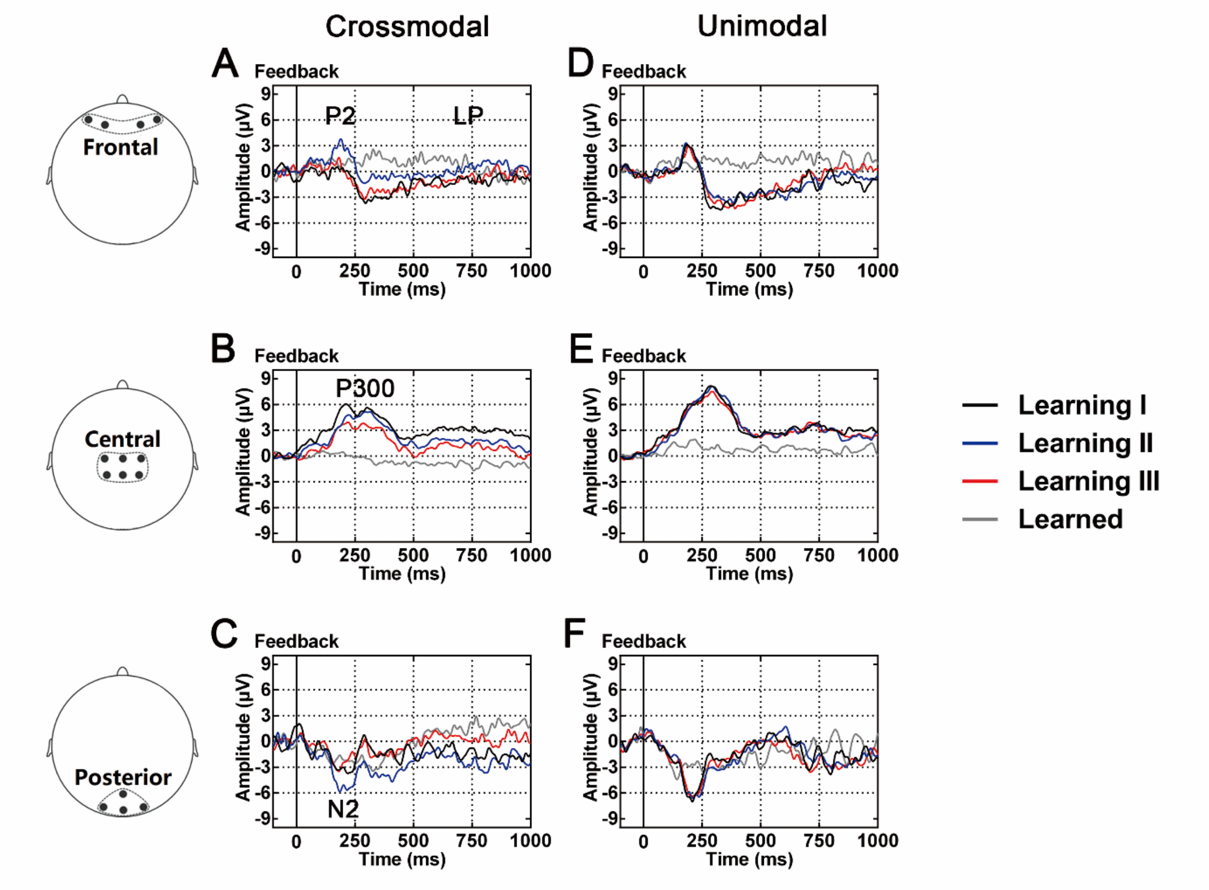

Supplementary Figure 6. Grand average ERPs recorded during the feedback period (between 100 ms before and 1000 ms after the onset of feedback) over frontal **(A, D)**, central **(B, E)** and posterior **(C, F)** recording sites for different learning sessions in the poor learners. ERP components including P2/N2 complex **(A, C)**, P300 **(B)** and late potential **(A)** are indicated in the figure.

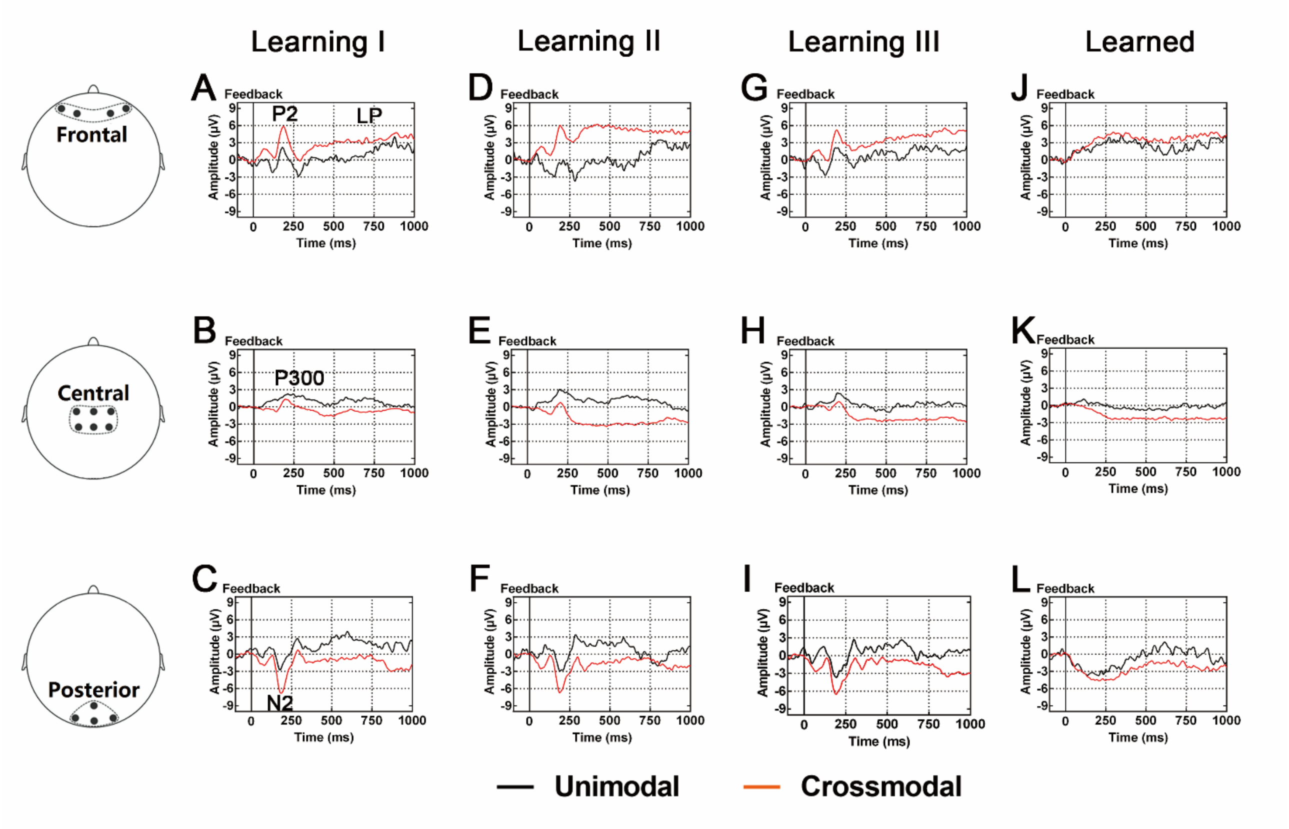

Supplementary Figure 7. Grand average ERPs recorded during the feedback period (between 100 ms before and 1000 ms after the onset of feedback) over frontal **(A, D, G, J)**, central **(B, E, H, K)** and posterior **(C, F, I, L)** recording sites for the crossmodal task and the unimodal task in the quick learners. ERP components including P2/N2 complex **(A, C)**, P300 **(B)** and late potential **(A)** are indicated in the figure.

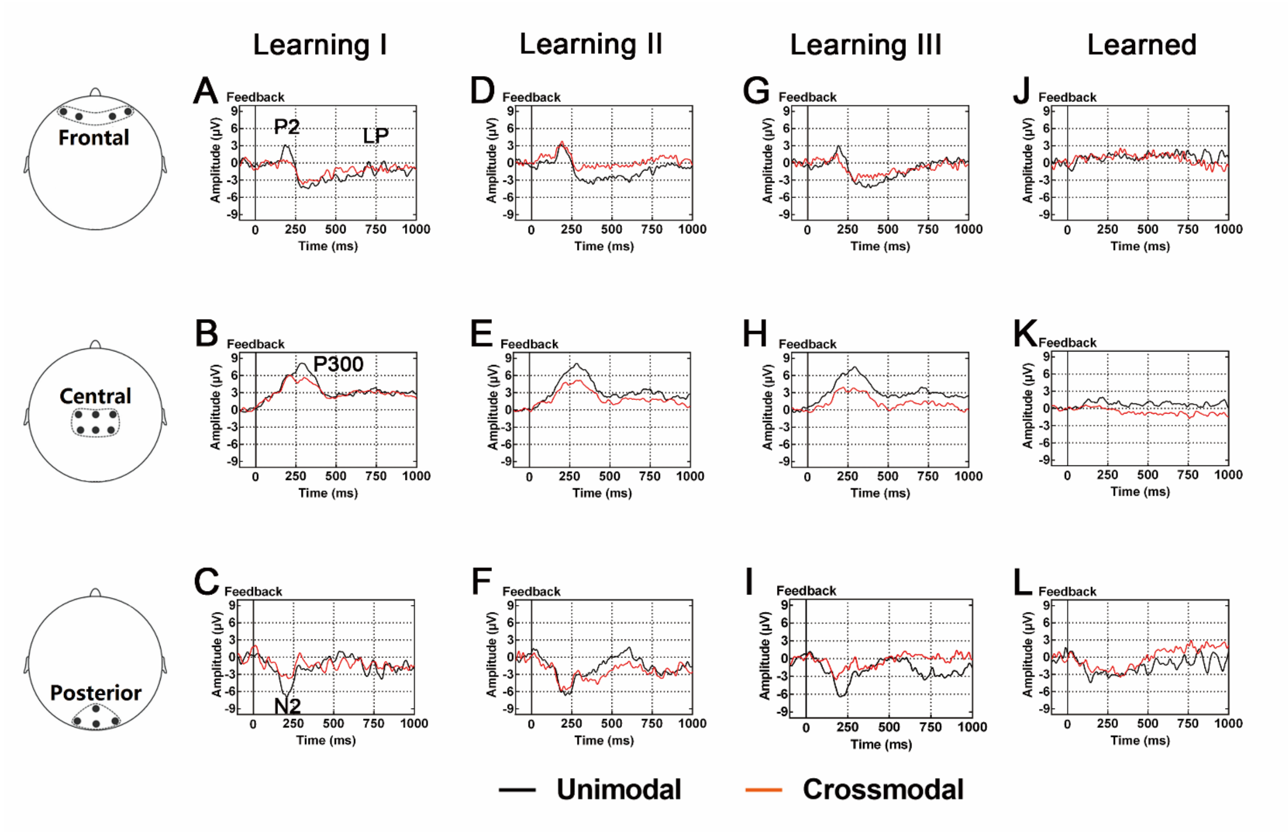

Supplementary Figure 8. Grand average ERPs recorded during the feedback period (between 100 ms before and 1000 ms after the onset of feedback) over frontal **(A, D, G, J)**, central **(B, E, H, K)** and posterior **(C, F, I, L)** recording sites for the crossmodal task and the unimodal task in the poor learners. ERP components including P2/N2 complex **(A, C)**, P300 **(B)** and late potential **(A)** are indicated in the figure.

1.2 Supplementary Table

Supplementary Table 1. The accuracy and reaction time for all nine blocks in three groups of participants.

			Block 1	Block 2	Block 3	Block 4	Block 5	Block 6	Block 7	Block 8	Block 9
Quick Learners	Crossmodal (n=7)	ACC (%)	85.12±2.20	92.86±2.68	95.83±1.57	98.21±1.24	100.00±0.00	96.43±0.60	100.00±0.00	98.81±0.77	97.62±0.84
		RT (ms)	662.35±63.38	572.04±61.05	489.28±58.60	437.71±67.63	411.57±81.34	400.53±60.45	427.29±74.46	438.74±61.76	431.20±61.63
	Unimodal (n=3)	ACC (%)	77.78±3.67	88.89±7.35	94.44±3.67	95.83±2.41	95.83±2.41	95.83±2.41	95.83±2.41	91.67±4.17	94.44±3.67
		RT (ms)	794.02±20.73	580.72±92.80	585.10±68.37	535.97±93.99	531.36±80.18	503.90±100.39	515.58±93.33	466.28±125.46	454.98±102.23
Good Learners	Crossmodal (n=16)	ACC (%)	56.77±2.11	71.88±2.93	83.59±3.28	88.54±2.76	90.36±2.54	91.15±1.82	92.71±2.59	92.71±1.72	94.27±2.31
		RT (ms)	857.46±62.83	806.85±64.15	689.34±64.09	626.08±68.38	587.87±77.48	529.72±59.05	548.70±52.56	579.30±74.50	552.01±67.50
	Unimodal (n=19)	ACC (%)	52.19±2.67	64.47±2.39	72.81±3.13	84.87±2.26	87.94±2.53	93.20±1.43	94.74±1.38	94.74±1.59	93.64±2.00
		RT (ms)	888.69±45.79	850.22±44.83	791.22±39.41	724.91±54.64	671.38±58.55	614.84±49.79	641.44±51.96	599.76±47.27	574.88±44.56
Poor Learners	Crossmodal (n=6)	ACC (%)	52.78±5.01	56.94±4.12	59.03±4.36	61.11±5.56	57.64±6.49	57.64±4.22	50.00±5.38	52.78±8.10	49.31±8.43
		RT (ms)	786.89±116.95	776.22±117.16	668.24±113.75	780.05±152.40	727.18±116.77	817.30±156.88	803.58±128.36	842.27±202.63	840.41±196.56
	Unimodal (n=6)	ACC (%)	52.78±6.05	58.33±4.03	57.64±6.40	56.94±2.98	63.89±1.76	64.58±6.34	55.56±6.43	61.81±1.99	54.86±3.94
		RT (ms)	984.43±51.24	990.46±88.41	888.16±61.22	922.56±45.01	901.38±53.92	852.51±63.13	779.70±121.36	767.21±116.47	772.06±120.51

ACC, accuracy.
